# Insulin-like growth factor-1 regulates the SIRT1-p53 pathway in cellular senescence

**DOI:** 10.1111/acel.12219

**Published:** 2014-04-30

**Authors:** Duc Tran, Johann Bergholz, Haibo Zhang, Hanbing He, Yang Wang, Yujun Zhang, Qintong Li, James L Kirkland, Zhi-Xiong Xiao

**Affiliations:** 1 Department of Biochemistry, Boston University School of MedicineBoston, MA, 02118, USA; 2 Center of Growth, Metabolism and Aging, Key Laboratory of Bio-Resource and Eco-Environment of Ministry of Education, College of Life Sciences and State Key Laboratory of Biotherapy, Sichuan UniversityChengdu, 610014, China; 3 Robert and Arlene Kogod Center on Aging, Mayo Clinic College of MedicineRochester, MN, 55905, USA

**Keywords:** aging, IGF-1, p53, senescence, SIRT1

## Abstract

Cellular senescence, which is known to halt proliferation of aged and stressed cells, plays a key role against cancer development and is also closely associated with organismal aging. While increased insulin-like growth factor (IGF) signaling induces cell proliferation, survival and cancer progression, disrupted IGF signaling is known to enhance longevity concomitantly with delay in aging processes. The molecular mechanisms involved in the regulation of aging by IGF signaling and whether IGF regulates cellular senescence are still poorly understood. In this study, we demonstrate that IGF-1 exerts a dual function in promoting cell proliferation as well as cellular senescence. While acute IGF-1 exposure promotes cell proliferation and is opposed by p53, prolonged IGF-1 treatment induces premature cellular senescence in a p53-dependent manner. We show that prolonged IGF-1 treatment inhibits SIRT1 deacetylase activity, resulting in increased p53 acetylation as well as p53 stabilization and activation, thus leading to premature cellular senescence. In addition, either expression of SIRT1 or inhibition of p53 prevented IGF-1-induced premature cellular senescence. Together, these findings suggest that p53 acts as a molecular switch in monitoring IGF-1-induced proliferation and premature senescence, and suggest a possible molecular connection involving IGF-1-SIRT1-p53 signaling in cellular senescence and aging.

## Introduction

The insulin-like growth factor (IGF) 1 and 2 proteins are evolutionarily conserved and play a central role in many cellular processes, including growth, proliferation, survival, development, and cancer (Maki, 2010). IGF-1-null, IGF-2-null, and even double-deficient mice are viable, although much smaller than wild-type counterparts, while IGF receptor 1 (IGFR1) knockout mice succumb to early postnatal lethality, indicating the importance of IGFR1 signaling for viability (Liu *et al*., 1993). IGF signaling plays a mitogenic role; activation of the IGF1R leads to upregulation of the PI3K/AKT pathway, thus increasing cell survival and promoting growth and proliferation (Blume-Jensen & Hunter, 2001). It has been shown that increased IGF signaling is associated with cancer progression (de Ostrovich *et al*., 2008). On the other hand, reduced IGF signaling is associated with increased longevity (Suh *et al*., 2008). Aging humans have been shown to accumulate senescent cells in multiple tissues (Jeyapalan *et al*., 2007). Cellular senescence is a process involving irreversible cell cycle arrest, thus opposing cancer progression and potentially playing a role in organismal aging. However, whether senescence is a required step in organismal, aging is still under much debate. Likewise, the role of IGFs in cellular senescence is largely unclear.

The NAD-dependent deacetylase SIRT1 has been shown to play an important role in organismal aging and cellular senescence. SIRT1 heterozygous MEF cells (Sirt1^+/−^) dramatically delay senescence compared with wild-type counterparts (Chua *et al*., 2005), and SIRT1 levels have been shown to be decreased in senescent cells (Michishita *et al*., 2005). Conversely, SIRT1 overexpression antagonizes oncogene-induced cellular senescence in human diploid fibroblasts (Huang *et al*., 2008), and inhibition of SIRT1 by sirtinol has been shown to induce senescence-like cell growth arrest in human breast cancer MCF7 and human lung adenocarcinoma H1299 cells (Ota *et al*., 2006). Moreover, Sir2, a yeast homolog of SIRT1, has been shown to be an important regulator of aging (Guarente & Kenyon, 2000), suggesting that SIRT1 may also be involved in aging and longevity. Importantly, SIRT1 has been shown to deacetylate and inactivate the tumor suppressor protein p53 (Langley *et al*., 2002).

The importance of p53 in cellular senescence has been demonstrated by studies showing accumulation of p53 in senescent cells (Atadja *et al*., 1995). Furthermore, activation of the p53 and Rb pathways via multiple mechanisms may directly lead to premature senescence (Kuilman *et al*., 2010). p53 levels and activation are tightly controlled under normal physiological conditions, but increase rapidly as a response to a diverse range of cellular stresses, which mediate a number of post-translational modifications, such as phosphorylation and acetylation, that affect p53 stability and function (Smeenk & Lohrum, 2010). For instance, the balance between acetylation by histone acetyl transferases (HATs) and deacetylation by histone deacetylases (HDACs) plays an important role in p53 activation and in discerning the specific cellular outcome of this activation (Smeenk & Lohrum, 2010). Importantly, p53 deacetylation by SIRT1 has been shown to be an important regulator of premature cellular senescence (Langley *et al*., 2002). Moreover, p53 has also been shown to be involved in organismal aging in mice bearing a mutant p53 allele that expresses a short p53 protein species with higher transactivation activity. These mice exhibit an accelerated aging phenotype, exemplified by kyphosis, osteoporosis, generalized organ atrophy, and diminished stress tolerance (Tyner *et al*., 2002).

In this study, we show that prolonged IGF-1 treatment leads to premature cellular senescence by activating the tumor suppressor protein p53. Importantly, we demonstrate that p53 possesses dual functions in response to IGF-1. While short-term IGF-1 induces cell proliferation that is antagonized by p53, long-term IGF-1 exposure activates p53, leading to premature cellular senescence. We show that IGF-1 inhibits SIRT1 deacetylase activity via PI3K signaling, resulting in increased acetylated p53 levels. Furthermore, ectopic expression of SIRT1 blocks IGF-1-induced senescence. Thus, IGF-1 can promote premature cellular senescence by modulating the SIRT1-p53 axis.

## Results

### Prolonged treatment with IGF-1 promotes cellular senescence-like phenotypes

**Figure 1:**
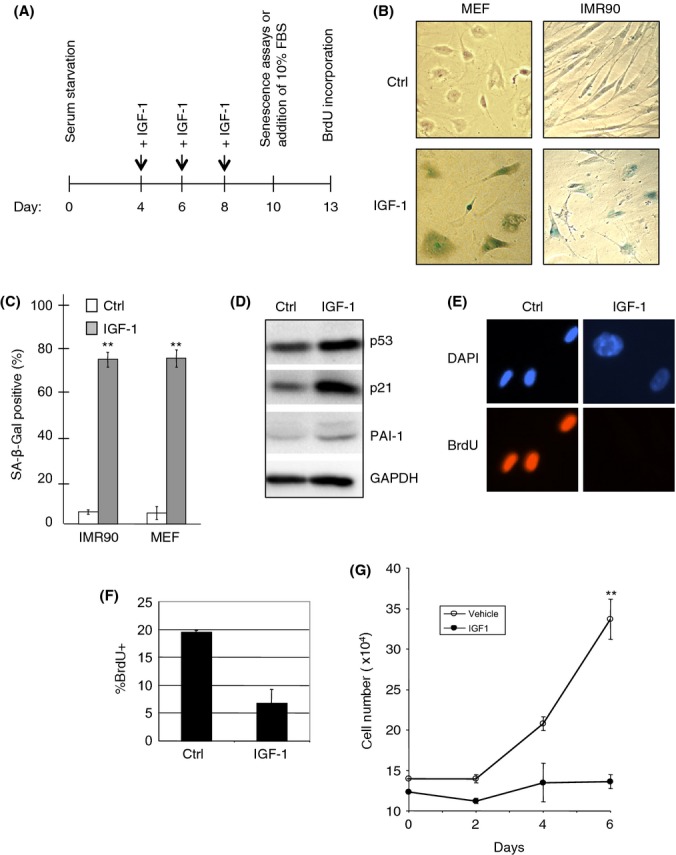
Prolonged insulin-like growth factor-1 (IGF-1) treatment induces senescence-like phenotypes. (A) Schematic of experimental design and reference time frame. (B, C) MEFs and IMR90 cells were serum-starved for 4 days, and then treated with or without 50 ng mL^−1^ IGF-1 for 6 days under serum-starvation conditions. Cells were stained for SA-β-Gal activity, photographed (B), and quantitated as the percentage of SA-β-Gal-positive cells over total cell number (C). Results are presented as means and SE from three experiments performed in triplicate. (D) IMR90 cells were serum-starved for 4 days, and then treated with or without IGF-1 for 6 days under serum-starvation conditions. Whole-cell lysates were subjected to western blot analysis, as shown. (E–G) IMR90 cells were serum-starved for 4 days, then treated with or without IGF-1 for 6 days under serum-starvation conditions, and then grown for an additional 3 days in the presence of 10% FBS. Cells were assayed for BrdU incorporation (E) and quantified (F), or grown for three more days in the presence of 10% FBS and measured for cell proliferation (G). Results are presented as means and SE from three experiments performed in triplicate. **P < 0.01.

IGF/insulin signaling is clearly implicated in organismal aging, yet the molecular basis for this regulation is not well understood. As a body of evidence indicates that cellular senescence contributes to aging phenotypes (Baker *et al*., 2011), we asked whether prolonged IGF-1 treatment could promote cellular senescence. Human primary fibroblast IMR90 cells and mouse embryonic fibroblasts (MEFs) were serum-starved for 4 days prior to treatment, as previously described (Serrano *et al*., 1997), to reduce any confounding variables by exogenous IGF-1 present in the serum used to supplement the growth media. Cells were treated with IGF-1 for six additional days and analyzed for senescence-associated phenotypes, as diagramed in Fig. 1(A). IGF-1 treatment of IMR90 or MEF cells led to appearance of cells with feature characteristic of premature cellular senescence, including an enlarged flat cell morphology and increased β-Galactosidase (SA-β-Gal) activity (Fig. 1B,C). In addition, we analyzed markers of senescence in IMR90 cells after prolonged IGF-1 treatment. We observed upregulation of p53 protein and of the cyclin-dependent kinase (CDK) inhibitor p21^CIP1^, a downstream target of p53, as well as increased plasminogen activator inhibitor-1 (PAI-1), a marker of senescence (Fig. 1D). Under conditions of serum depletion, we observed a minimal number of apoptotic cells, which was marginally affected by IGF-1 (Fig. S1A).

To determine whether IGF-1-treated cells exhibited irreversible cell growth arrest, cells were serum-starved for 4 days prior to treatment with or without IGF-1 for an additional 6 days, and then subjected to growth stimulation by supplementing the media with 10% FBS, followed by monitoring cell number and BrdU incorporation. As shown in Fig. 1(E,F), control IMR90 cells exhibited significant BrdU incorporation (20 ± 1%) and normal fibroblastic cell morphology upon serum stimulation. By contrast, cells treated with IGF-1 remained morphologically large and flat, with marginal BrdU incorporation (6 ± 3%). Control IMR90 cells, but not IGF-1-treated cells, continued to grow exponentially after serum reconstitution (Fig. 1G). These results indicate prolonged IGF-1 treatment of serum-starved cells can induce premature cellular senescence.

### IGF-1 induces premature senescence in a p53-dependent manner

**Figure 2:**
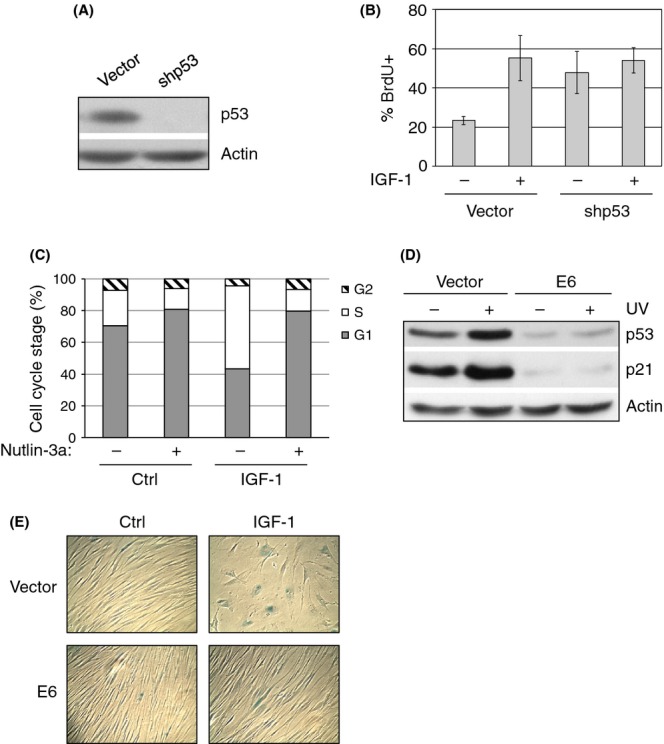
Insulin-like growth factor-1 (IGF-1) induces premature senescence in a p53-dependent manner. (A) MCF7 cells were stably infected with retrovirus expressing shRNA specific for p53 or a vector control. Cell lysates were subjected to western blotting as indicated. (B) MCF7 cells were serum-starved in the absence of FBS for 48 h, then treated with 50 ng mL^−1^ IGF-1 for 24 h prior to BrdU incorporation assay, as described in the Materials and Methods. BrdU incorporation was quantified as the percentage of BrdU-positive (% BrdU+) cells over the total number of cells. Results are presented as means and SE from three experiments. (C) MCF7 cells were serum-starved for 48 h and then treated with 50 ng mL^−1^ IGF-1 and/or 5.0 nm Nutlin-3a for 24 h. Cells were then analyzed by flow cytometry. Results are presented as means from two experiments. (D) IMR90 cells stably expressing E6 or vector control were UV irradiated. Cell lysates were subjected to western blot analysis as shown. (E) Stable IMR90 cells were serum-starved for 4 days and then treated with or without IGF-1 for 6 days under serum-starvation conditions and assayed for SA-β-Gal activity. Micrographs are representative of three independent experiments.

We showed that prolonged IGF-1 treatment of serum-starved primary fibroblasts leads to premature cellular senescence. Notably, IGF-1 has been shown to promote cell proliferation (Maki, 2010). Because the tumor suppressor protein p53 plays a critical role in cellular response to stress signals including abnormal oncogenic stress, we examined the role of p53 in IGF-1-induced cell proliferation. MCF7 cells were infected with retrovirus encoding p53 shRNA (Fig. 2A), followed by 24 h of serum starvation prior to IGF-1 treatment for 24 h. Cells were then subjected to BrdU incorporation assays. Short-term IGF-1 treatment induced BrdU incorporation (Fig. 2B). Notably, p53 silencing led to comparable increases in BrdU incorporation compared with IGF-1 treatment alone, while additional IGF-1 treatment did not further increase BrdU incorporation in p53-ablated cells (Fig. 2B). To demonstrate that acute IGF-1 treatment induces cellular proliferation and that this is counteracted by p53, we treated serum-starved MCF7 cells with IGF-1 in the presence or absence of the pharmacological p53 activator Nutlin-3a and analyzed cells by flow cytometry. As shown in Fig. 2(C), whereas serum starvation resulted in G1 cell cycle arrest, IGF-1 treatment promoted cell cycle progression, as indicated by a large increase in the percentage of cells in S-phase. Nutlin-3a abrogated the effect of IGF-1 in cell cycle progression, thus maintaining cells predominantly in G1 phase. These data indicate that IGF-1 promotes cell cycle progression of growth-arrested cells, which is inhibited by activation of p53.

As p53 plays an important role in oncogene-induced premature cellular senescence (Kuilman *et al*., 2010), we examined whether p53 is involved in IGF-1-induced premature cellular senescence. IMR90 cells stably expressing HPV E6 (E6), which effectively degraded p53 even upon DNA damage (Fig. 2D), were subjected to prolonged IGF-1 treatment. While IGF-1 induced SA-β-Gal activity and a large and flat cell morphology in control IMR90-vector cells, IGF-1 was unable to induce these phenotypes in IMR90–E6 cells (Fig. 2E). In addition, IGF-1 can also induce premature cellular senescence in cancer cells that contain wild-type p53, such as MCF7 and HCT116 (Fig. S1B,C). A high percentage of HCT116(p53^+/+^) cells treated with prolonged IGF-1 displayed senescence-associated phenotypes. By contrast, IGF-1 did not significantly induce premature cellular senescence in p53-null HCT116 cells (Fig. S1B,C). These results suggest that p53 represses short-term IGF-1 mitogenic function and is necessary for prolonged IGF-1 treatment-mediated induction of premature cellular senescence upon serum deprivation.

### IGF-1 stabilizes and activates p53

**Figure 3:**
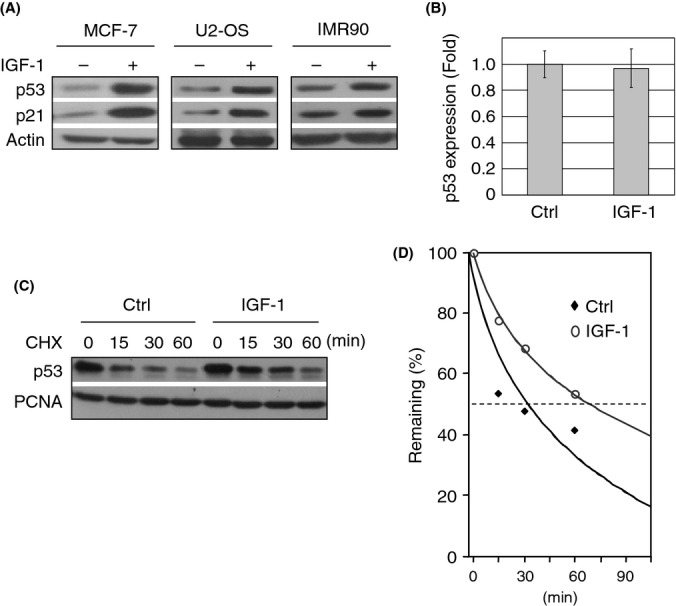
Insulin-like growth factor-1 (IGF-1) stabilizes and activates p53. Subconfluent cells were serum-starved for 48 h and treated with 50 ng mL^−1^ IGF-1 for 12 h. (A) Cell lysates from MCF7, U2-OS, and IMR90 cells were subjected to western blot analysis. (B) Serum-starved and IGF-1-treated MCF7 cells were subjected to Q-PCR analysis. Three independent experiments in duplicate were performed. (C) Serum-starved and IGF-1-treated MCF7 cells were treated with cyclohexamide (CHX). Cell lysates collected at different time intervals were subjected to immunoblotting, as indicated. Figure shown is representative of three independent experiments. (D) Relative p53 protein levels were quantified by densitometry.

Because p53 is necessary for IGF-1-mediated induction of cellular senescence, we investigated the underlying molecular mechanisms. As shown in Fig. 3(A), IGF-1 treatment led to a marked increase in p53 protein levels in serum-starved MCF7, U2-OS, and IMR90 cells, concomitant with an increase in p21^CIP1^ expression. Quantitative PCR analysis showed that IGF-1 did not alter p53 mRNA levels (Fig. 3B). Therefore, we examined the effect of IGF-1 on p53 protein stability. As shown in Fig. 3(C,D), IGF-1 treatment led to a substantial increase in p53 protein half-life, compared with the control. These data suggest that IGF-1 upregulates p53 at the post-translational level by inducing stabilization of the p53 protein.

### IGF-1 inhibits SIRT1 and induces p53 acetylation in a PI3K-dependent manner

**Figure 4:**
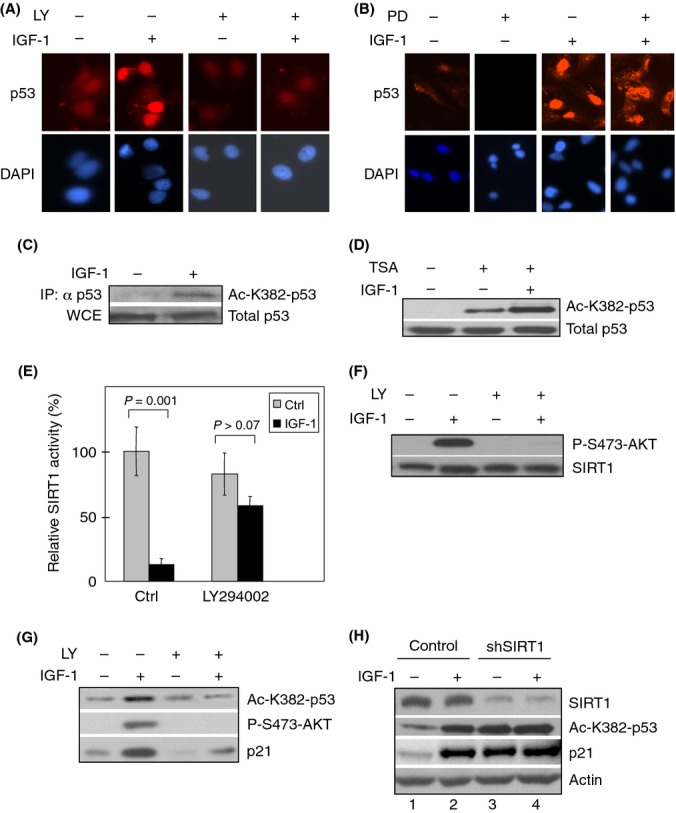
Insulin-like growth factor-1 (IGF-1) inhibits SIRT1 and induces p53 acetylation in a PI3K-dependent manner. (A, B) Serum-starved MCF7 cells were pretreated for 4 h with the PI3K inhibitor LY294002 (LY; 25 μm), the Mek-Erk inhibitor PD98059 (PD; 25 μm) or DMSO as a vehicle control. Cells were then treated with 50 ng mL^−1^ IGF-1 for 12 h. Cells were subjected to immunostaining for p53 and counterstained with DAPI. (C) Serum-starved MCF7 cells were treated with IGF-1 for 12 h. Whole-cell extract (WCE) were immunoblotted using a pan-specific antibody for p53. Normalized WCE containing comparable amount of total p53 protein were immunoprecipitated and immunoblotted with an antibody specific for acetylated p53 (Ac-K382-p53). (D) MCF7 cells were serum-starved for 48 h and treated with IGF-1 for 12 h. Cells were then treated with 40 μm Trichostatin A (TSA) for 6 h before collection. Whole-cell extracts were subjected to western blotting as indicated. (E–G) Subconfluent MCF7 cells were serum-starved for 48 h and pretreated for 2 h with 25 μm LY294002 or vehicle (Ctrl), prior to treatment with IGF-1 for 12 h. (E) Equal amounts of whole-cell extracts were subjected to SIRT1 immunoprecipitation for *in vitro* Fluor de Lys (Biomol) deacetylation assay. Relative SIRT1 activity is presented as means and SE from two independent experiments performed in duplicate. (F) Western blot analyses were performed to ensure comparable SIRT1 protein input from IP for Fluor de Lys assay and for analysis of IGF-1-induced AKT activation. (G) Whole-cell extracts were subjected to western blot analysis as shown. (H) MCF7 cells were stably infected with retrovirus expressing shRNA specific for SIRT1 or a control and selected by puromycin resistance. Cells were serum-starved for 48 h and treated with 50 ng mL^−1^ IGF-1 for 12 h prior to treating with 40 μm Trichostatin A for 6 h. Whole-cell extracts were subjected to western blotting.

IGF-1-mediated p53 protein accumulation was recapitulated in immunostained cells (Fig. 4A). Furthermore, pharmacological inhibition of PI3K by LY294002 abrogated IGF-1-mediated p53 upregulation (Fig. 4A), while inhibition of the MAP kinase pathway by PD98059 did not reduce IGF-1-mediated activation of p53 (Fig. 4B). These data suggest that IGF-1 activates p53 in serum-starved cells through the PI3-kinase cascade, in line with previous observations that IGF-1 modulates p53 protein levels and activities via several different mechanisms (Levine *et al*., 2006).

Acetylation is an important regulatory mechanism in the activation of p53 upon cellular stresses such as oncogenic stress (Lin *et al*., 1998)**.** Several HATs and HDACs can modify the acetylation of K382 on p53 (Sakaguchi *et al*., 1998). We therefore examined the effect of IGF-1 on p53 acetylation. Serum-deprived MCF7 cells were treated with IGF-1 for 12 h. As shown in Fig. 4(C), immunoprecipitation of total p53 followed by immunoblotting against acetylated p53 on K382 (ac-K382) indicated that IGF-1 significantly enhanced p53 K382 acetylation. In addition, IGF-1 was able to stimulate p53 acetylation despite the presence of trichostatin A (TSA; Fig. 4D). As TSA inhibits all members in the HDAC-classes I, II, and IV families, but not the sirtuin family (HDAC-class III), these data suggest that IGF-1 stimulates acetylation of K382 on p53 in an HDAC-class I/II/IV-independent manner.

We then examined the effect of IGF-1 on SIRT1, which is known to deacetylate and inhibit p53 (Langley *et al*., 2002). Serum-starved MCF7 cells were treated with or without IGF-1 prior to SIRT1 immunoprecipitation, which was then subjected to a deacetylation activity assay. As shown in Fig. 4(E), IGF-1 significantly inhibited SIRT1 deacetylase activity. We confirmed comparable SIRT1 protein input levels by western blot analysis (Fig. 4F). In addition, IGF-1 led to AKT activation, as evidenced by increased AKT phosphorylation (Fig. 4F). Thus, these data suggest that IGF-1 may exert these effects on p53 function via inhibition of SIRT1 activity. Furthermore, because our results showed that IGF-1-mediated upregulation of p53 in serum-starved cells requires PI3K activity, we examined whether PI3K is also necessary for IGF-1-mediated inhibition of SIRT1 activity. Pretreatment of serum-starved cells with LY294002 prior to IGF-1 treatment completely abrogated IGF-1-mediated upregulation of phospho-AKT, and it also effectively released IGF-1-mediated repression of SIRT1 deacetylase activity (Figs. 4E,F). These data suggest that IGF-1-mediated SIRT1 repression requires PI3K activity.

Next, we investigated the effect of PI3K inhibition on IGF-1-mediated induction of p53 acetylation. MCF7 cells were treated with or without IGF-1 in the presence or absence of LY294002. As shown in Fig. 4(G), IGF-1 stimulated p53 K382 acetylation, concomitant with p21^CIP1^upregulation, while treatment with LY294002 abrogated IGF-1-induced p53 acetylation. To investigate the specific role of SIRT1 in IGF-1-mediated upregulation of p53 acetylation, we generated stable MCF7 cells that expressed shRNA specific for SIRT1 or a control. We treated these cells with or without IGF-1 in the presence of TSA to eliminate the impact of members of other HDAC classes on p53 K382 acetylation. As shown in Fig. 4(H), in serum-starved cells, silencing of SIRT1 led to upregulation of acetylated p53 to levels similar to those in IGF-1-treated control cells (compare lanes 2 and 3), suggesting that IGF-1 specifically inhibits SIRT1 deacetylase activity toward p53. Moreover, IGF-1 did not further enhance p53 acetylation in the absence of SIRT1 (compare lanes 3 and 4), suggesting that IGF-1 treatment modulates p53 K382 acetylation primarily through inhibition of SIRT1, but not other HDAC proteins or through activation of HATs.

### SIRT1 attenuates IGF-1 induction of premature cellular senescence

**Figure 5:**
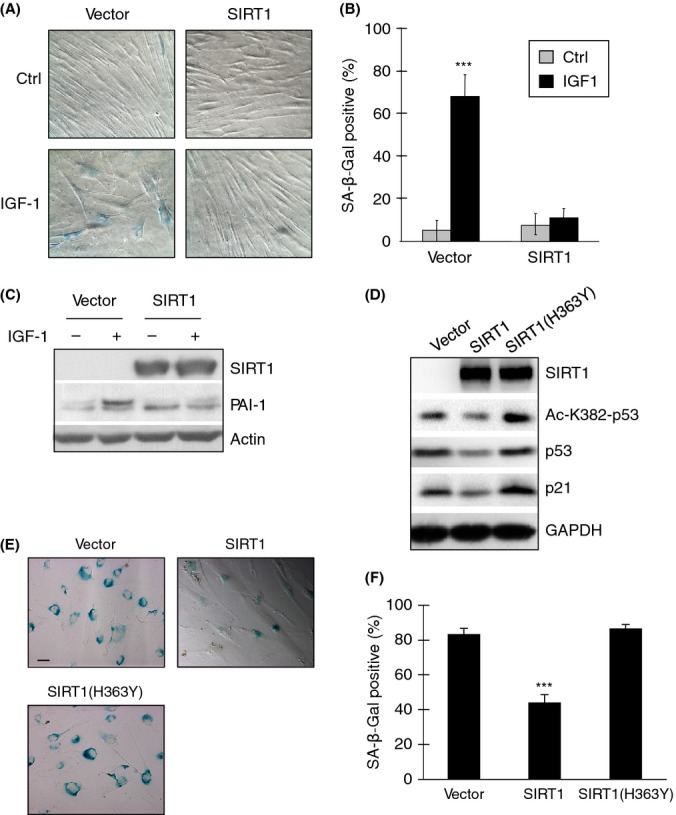
Ectopic expression of SIRT1 attenuates insulin-like growth factor-1 (IGF-1)-induced premature cellular senescence. IMR90 cells were infected with retrovirus expressing murine SIRT1 or vector control and selected by puromycin resistance. Stable cells were serum-starved for 4 days and then treated with 50 ng mL^−1^ IGF-1 every other day for 6 days. (A) Cells were assayed for SA-β-Gal activity and photographed under a light microscope. (B) Senescent cells were quantified by counting the number of SA-β-Gal-positive cells over the total number of cells from four randomly selected fields of each cell culture plate. Results are presented as means and SE from two experiments performed in duplicate. (C) Whole-cell extracts were subjected to western blot analysis as shown. (D–F) IMR90 cells were stably transfected with lentivirus expressing human wild-type SIRT1 or point-mutant SIRT1(H363Y), or a vector control. Stable cells were serum-starved for 4 days and then treated with 50 ng mL^−1^ IGF-1 every other day for 6 days. (D) Whole-cell lysates were subjected to western blot analysis. (E) Stable cells were assayed for SA-β-Gal activity and photographed under a light microscope. (F) Senescent cells were quantified as before. Results are presented as means and SE from two experiments. ***P < 0.001.

We next examined the importance of SIRT1 in IGF-1-induced premature cellular senescence. IMR90 cells stably expressing SIRT1 or vector were serum-starved and treated with or without prolonged IGF-1. As shown in Fig. 5(A–C), cells expressing the vector control became senescent upon IGF-1 treatment, as evidenced by large and flat cell morphology, SA-β-Gal staining, and PAI-1 upregulation. By contrast, cells overexpressing SIRT1 did not exhibit senescence-associated phenotypes, in spite of the presence of IGF-1. Notably, SIRT1-overexpressing cells exhibited only basal levels of SA-β-Gal staining that were unaffected by IGF-1 treatment (Fig. 5A,B). In addition, SIRT1 overexpression abrogated IGF-1-mediated upregulation of PAI-1 (Fig. 5C). To further demonstrate that SIRT1 deacetylase activity is essential in regulating IGF-1-induced cellular senescence, we expressed a point-mutant human SIRT1(H363Y) that is incapable of deacetylating p53, as shown previously (Luo *et al*., 2001). Upon prolonged IGF-1 treatment, wild-type SIRT1 expression resulted in decreased p53 acetylation at K382 and reduced p21^CIP1^ expression, while SIRT1(H363Y) expression did not significantly affect p53 acetylation or p21^CIP1^ expression (Fig. 5D). Moreover, while wild-type SIRT1 overexpression consistently inhibited prolonged IGF-1-induced premature cellular senescence in IMR90 cells, SIRT1(H363Y) was unable to do so (Fig. 5E,F). Taken together, these data show that SIRT1 deacetylase activity toward p53 plays a critical role in IGF-1-mediated premature cellular senescence.

Furthermore, we investigated the molecular mechanisms by which IGF-1 inhibits SIRT1 deacetylase activity. It has been shown that the protein deleted in breast cancer 1 (DBC1) can inhibit SIRT1 activity, including its ability to deacetylate p53 (Kim *et al*., 2008). Therefore, we examined whether IGF-1 affects the SIRT1-DBC1 interaction under our experimental settings. We treated MCF7 cells with or without IGF-1 and then performed IP assays to examine SIRT1–DBC1 interaction. We did not detect significant changes in SIRT1–DBC1 interaction between cells treated with IGF-1 and control cells (Fig. S2A,B), indicating that IGF-1-induced inhibition of SIRT1 deacetylase activity toward p53 is not likely to be mediated by DBC1. Furthermore, while IMR90 cells can be induced to premature cellular senescence by IGF-1, DBC1 protein levels are hardly detectable by western blotting in these cells (Fig. S2C). These data suggest that DBC1 is unlikely to play a major role in IGF-1-induced premature cellular senescence.

## Discussion

**Figure 6:**
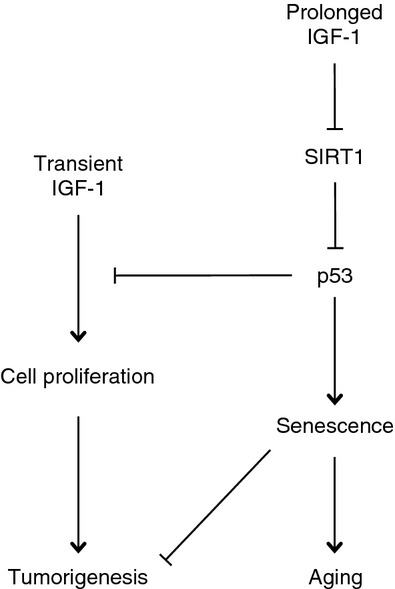
A working model for insulin-like growth factor-1 (IGF-1) dual function and p53 acting as a molecular switch. IGF-1 can function as a mitogen to promote cell proliferation and survival, whereas it can also promote cellular senescence. In the latter case, prolonged IGF-1 leads to inhibition of SIRT1 deacetylase activity. Reduced SIRT1 activity results in increased p53 acetylation on K382, thus leading to p53 activation, which in turn upregulates p21^CIP1^, thereby promoting cell growth arrest and cellular senescence. Premature cellular senescence inhibits tumorigenesis and promotes organismal aging.

Insulin and insulin-like growth factors play an important role in carcinogenesis and longevity. In this study, we demonstrate that IGF-1 exerts a dual mode of action in growth stimulation and growth inhibition. Namely, while acute IGF-1 exposure leads to increased cellular proliferation, prolonged IGF-1 treatment promotes premature cellular senescence upon serum deprivation. Our results show that p53 is pivotal in mediating the dual functions of IGF-1. IGF-1 promotes proliferation and survival, both of which are monitored by p53 (Murray *et al*., 2003). On the other hand, prolonged IGF-1 treatment inhibits SIRT1 deacetylase activity, thus resulting in increased p53 acetylation and transactivation activity, leading to premature cellular senescence in serum-starved cells. Therefore, p53 can modulate the dual functions of IGF-1 by acting as a molecular switch (Fig. 6).

It is well documented that IGF-1 functions as a mitogen. However, whether IGF-1 induces cellular senescence is largely unknown. It has been shown that various oncogenic or mitogenic stresses can induce premature cellular senescence. For example, abnormal activation of proto-oncogenes such as c-myc, *Ras*, STAT5A, and E2F1, or expression of viral oncogenes such as E1A, are capable of inducing premature cellular senescence (Courtois-Cox *et al*., 2008). Although initially mitogenic, hyperactivated *Ras* effectively promotes oncogene-induced cellular senescence at a later stage by inducing p16^INK4A^ as well as ARF, which inhibit MDM2, leading to increased p53 proteins levels (Lin *et al*., 1998). Likewise, we found that IGF-1 treatment initially promotes proliferation and later induces senescence in a SIRT1/p53-dependent manner. It is conceivable that strength and duration of IGF-1 signaling is important in affecting the kinetics of SIRT1-dependent p53 deacetylation, activation and function.

DNA damage can also induce premature senescence. For example, ionizing radiation of human fibroblasts has been shown to induce p53 and senescence *in vitro* (Di Leonardo *et al*., 1994) and *in vivo*, exemplified by administration of cyclophosphamide to mice bearing tumor xenografts (Schmitt *et al*., 2002). Furthermore, premature senescence can be induced by increased production of reactive oxygen species (ROS; Lu & Finkel, 2008). Notably, oncogene-induced premature senescence can also invoke ROS, leading to activation of p53 (Moiseeva *et al*., 2009). It has been shown that reduced IGF-1 signaling protects against oxidative damage in simple organisms and in mice (Holzenberger *et al*., 2003), likely through reduced AKT activity, which is known to promote ROS production (Di Segni *et al*., 2006). As ROS are known inducers of cellular senescence (Lu & Finkel, 2008), it is conceivable that IGF-1 increases ROS, leading to cellular senescence. Although we did not specifically examine oxidative stress in our study, it is possible that ROS play a role in IGF-1-mediated induction of premature cellular senescence.

In addition to nontransformed cells such as IMR90, IGF-1 can also induce premature senescence in cancer cells that contain wild-type p53. Our results indicate that p53 plays a critical role in IGF-1-induced premature cellular senescence, as inhibition of p53 by E6 completely blocks this process. We show that IGF-1 induces p53 acetylation in a SIRT1-dependent manner, leading to activation of p53 transactivation activity and premature senescence. These observations are in line with reports linking p53 to organismal aging, suggesting that p53 may be a link between cellular senescence and aging. Indeed, transgenic mice bearing hyperactive p53 alleles exhibit increased circulating levels of IGF-1 and accelerated aging phenotypes (Tyner *et al*., 2002; Maier *et al*., 2004). However, whether p53 drives organismal aging is still under debate. Mice bearing an extra copy of the p53 gene have been independently reported to either exhibit accelerated aging or to age normally (García-Cao *et al*., 2002; Tyner *et al*., 2002; Maier *et al*., 2004). In addition, mice with hypomorphic mdm2 alleles, resulting in higher p53 protein expression, do not exhibit progerias. In these cases, total p53 protein levels were increased, yet nuclear p53 levels were unchanged (Mendrysa *et al*., 2006). These observations suggest that activation of p53 is necessary but not sufficient for organismal aging, which is regulated by a complex network involving growth, stress, and hormonal signaling among others.

Here, we show that IGF-1 induces specific p53 acetylation via inhibition of SIRT1, leading to premature senescence. Importantly, ectopic expression of SIRT1 effectively abrogates IGF-1-induced cellular senescence, thus linking the IGF-1-SIRT1-p53 pathway to cellular senescence and possibly aging. Interestingly, it has been shown that IGF-1 attenuates calorie restriction-induced SIRT1 expression, yet the molecular mechanisms by which IGF-1 regulates SIRT1 are still unclear (Cohen *et al*., 2004). Our results indicate that IGF-1 significantly inhibits SIRT1 deacetylase activity, yet it does not alter SIRT1 protein levels. Therefore, it is plausible that IGF-1 may inhibit SIRT1 activity by other means. Notably, IGF-1 has been shown to inhibit AMPK activity via AKT-dependent phosphorylation (Ning *et al*., 2011). AMPK, on the other hand, can increase cellular NAD+ levels in mouse skeletal muscle via mitochondrial-mediated β-oxidation, thus leading to enhanced SIRT1 deacetylation activity (Canto *et al*., 2009). However, we found that immunoprecipitated SIRT1 proteins derived from IGF-1-treated cells exhibited reduced deacetylase activity *in vitro*, compared with untreated cells even in the presence of equal NAD+ concentrations, thus suggesting that IGF-1-mediated regulation of SIRT1 is independent of NAD+. Alternatively, IGF-1 may directly or indirectly induce SIRT1 post-translational modifications, thereby modulating its activity. It was shown that AMPK can directly phosphorylate SIRT1 on T344, thereby inhibiting SIRT1 activity in human hepatocellular carcinoma cells (Lee *et al*., 2012). Clearly, regulation of SIRT1 activity and p53 acetylation is highly cell type and context-dependent, and it is therefore plausible that IGF-1 inhibits SIRT1 via AMPK or by other means.

Hence, it is conceivable that cellular senescence induced by the IGF-1-SIRT1-p53 axis contributes to the senescence and aging processes *in vivo*. Mice with reduced IGF-1 signaling (IGF-1 receptor heterozygous knockout or PAPP-A knockout mice) exhibit both delay in age-related senescent cell accumulation and prolonged lifespan (Holzenberger *et al*., 2003; Conover & Bale, 2007). Transgenic mice expressing bovine growth hormone, which leads to increased IGF-1 levels, have an accelerated aging phenotype (Ding *et al*., 2013). Moreover, IGF-1 receptor polymorphisms, which result in impaired receptor function, are present more frequently in human centenarians than in the general population (Suh *et al*., 2008). In rodents, long-term caloric restriction is associated with decreases in circulating IGF-1 by up to 50% (Dunn *et al*., 1997), thus further implicating IGF signaling with the aging process (Flurkey *et al*., 2001). In addition, caloric restriction is associated with reduced abundance of senescent cells in the small intestine in mice (Wang *et al*., 2010). Importantly, clearance of senescent cells from progeroid mice delays phenotypic characteristics of aging and extends healthspan (Baker *et al*., 2011). Moreover, we showed that PI3K signaling is critical for mediating the effects of IGF-1 on cellular senescence, because pharmacological inhibition using LY294003 completely abrogates these effects. This further supports a link between cellular senescence and organismal aging, as mutations on the *C. elegans* ortholog of the catalytic subunit of PI3K, *age-1*, lead to lifespan extension (Friedman & Johnson, 1988).

Our study strongly suggests that the IGF-1-SIRT1-p53 pathway plays an important role in regulating cellular senescence. Taken together, these studies strongly implicate IGF-1 signaling in senescence and aging.

## Experimental procedures

### Cell culture and IGF-1 treatment

U2-OS, MCF7, IMR90, HCT116, A549, and MEF cells were maintained in DMEM supplemented with 10% (12% for MEFs) FBS and 1% penicillin/streptomycin sulfate (Invitrogen Inc., Carlsbad, CA, USA) at 37 °C in a humidified incubator under 5% CO_2_. Prior to IGF-1 treatment, MCF7 and HCT116 cells at 60–70% confluency were incubated in serum-free DMEM for 48 h, while MEFs and IMR90 cells were serum-starved in DMEM containing 0.5% FBS. IGF-1 (human recombinant; Sigma, St. Louis, MO, USA) was prepared as a 100 μg mL^−1^ stock solution in PBS according to manufacturer’s instructions. LY294002 (Calbiochem, San Diego, CA, USA) was prepared as a 25 mm stock solution. Trichostatin A (TSA; Sigma) was prepared as a 2 mg mL^−1^ stock in DMSO. At 45 min prior to IGF-1 treatment, LY294002 was added to serum-starved cells at a final concentration of 25 μm. For acetylation of endogenous p53, cells were deprived of serum for 48 h before treatment with IGF-1 for 12 h and subsequently treated with 40 μm Trichostatin A for 6 h. Nutlin-3a (Sigma) was used at 5.0 nm either with IGF-1 or with the vehicle control.

### Assays for senescence-associated phenotypes

Assays for assessing cellular senescence were performed essentially as described (Serrano *et al*., 1997). Briefly, cells were serum-starved for 96 h prior to treatment with 50 ng mL^−1^ IGF-1 or vehicle. Media (DMEM containing either no FBS or 0.5% FBS) supplemented with fresh IGF-1 were replaced every 48 h. Six days after IGF-1 treatment, cells were either subjected to morphological examination or stained for senescence-associated β-Galactosidase (SA-β-Gal), for which cells were washed with PBS (pH 7.2), fixed with 0.4% paraformaldehyde in PBS, pH 7.4, and stained in X-Gal solution for 16 h at 37 °C. Cells were then visualized under a light microscope and assessed for percentage of large, flat cell morphology and SA-β-Gal activity. To assay for growth arrest, 6 days after IGF-1 treatment, cells were refed with 10% FBS/DMEM for 3 days and subjected to growth curve determination or 5-bromo-2′-deoxyuridine (BrdU) incorporation assay.

### BrdU incorporation and immunofluorescence

For BrdU incorporation assay, cells were labeled with 10 μm BrdU (Roche, Indianapolis, IN, USA) for 2 h, fixed with 4% para-formaldehyde, and immunostained with anti-BrdU antibody (Roche) followed by staining with Cy™3-conjugated goat anti-mouse IgG (115-165-146; Jackson ImmunoResearch Laboratories, West Grove, PA, USA) and counter-stained with DAPI. BrdU-positive cells were scored under a fluorescent microscope and presented as the percentage of BrdU-positive nuclei over total number of nuclei counted. At least 300 nuclei were counted. For immunofluorescence, cells were fixed with 4% paraformaldehyde, immunostained with primary and secondary antibodies in 4% BSA, and counter-stained with DAPI. Antibodies used include anti-p53 (DO-1; Santa Cruz Biotechnology, Santa Cruz, CA, USA) and goat anti-mouse Alexa Fluor 488 (A11001; Santa Cruz Biotechnology). Cell images were recorded with an Axiovert 200M microscope (Carl Zeiss, Oberkochen, Germany) and analyzed with axiovision 3.1 software (Carl Zeiss).

### Flow cytometry analysis

Cells were trypsinized, washed with PBS, and fixed in 70% ethanol at 4 °C overnight. Cells were stained with propidium iodide (PI) supplemented with 100 μg mL^−1^ RNase A at 37 °C in dark for 1 h. Cells were subjected to flow cytometry analysis by FACScan Flow Cytometer (Becton Dickinson, Franklin Lakes, NJ, USA), and data were analyzed using cell quest software (Becton Dickinson).

### Viral infection and RNA interference and quantitative PCR

Cells were infected with retrovirus and selected by puromycin resistance (5 μg mL^−1^; Sigma). Retroviral-based expression plasmids include pBabe-E6 and pSuper-murine SIRT1, and retroviral-based short hairpin RNA expression plasmids include pSuper-shSIRT1 and pSuper-shp53. Human wild-type and mutant SIRT1 expression plasmids were subcloned into pLVX.puro lentiviral plasmids. All plasmids were confirmed by DNA sequencing. For RNA interference, cells were transfected with 200 nm p53 siRNA (Ambion) or control nonspecific siRNA (Dharmacon, Lafayette, CO, USA) using Lipofectamine 2000 (Invitrogen Inc., Carlsbad, CA, USA) or Fugene 6 (Roche). RNA was isolated using TRIzol (Gibco Life Technologies, Rockville, MD, USA) according to the manufacturer’s protocol and reverse transcribed using iScript cDNA synthesis kit (Bio-Rad, Hercules, CA, USA). Q-PCRs were performed in an ABI PRISM 7000 Sequence Detection System (Applied Biosystems, Foster City, CA, USA) using QuantiTect SYBR Green PCR Kit (Qiagen, Chatsworth, CA, USA) according to the manufacturer’s instructions.

### Western blot analyses and immunoprecipitation

For western blotting, cells were lyzed in EBC_250_ lysis buffer. Whole-cell lysates were separated by SDS–PAGE, transferred to PVDF membranes, and hybridized to an appropriate primary antibody and HRP-conjugated secondary antibody for subsequent detection by ECL. Antibodies used include actin (C-11, Santa Cruz Biotechnology), Ac-p53-K382 (2525P; Calbiochem, La Joya, CA, USA), GFP (FL; Santa Cruz Biotechnology), p21^CIP1^ (SXM-30; BD Pharmingen, San Diego, CA, USA), p53 (DO-1, Santa Cruz Biotechnology), phospho-Akt (Cell Signaling Technology, Danvers, MA, USA), SIRT1 (07-131, Upstate Biotechnology, Lake Pacid, NY, USA), and PAI-1 (D9C4, Cell Signaling Technology). For IP analyses, cells were lyzed in EBC_150_ buffer. Whole-cell lysates were precleared with protein A bead slurry (Upstate Biotechnology), incubated with an appropriate antibody at 4 °C overnight, and subsequently captured with protein A bead slurry (Upstate Biotechnology) for 3 h. Immunoprecipitates were subjected to western blot analyses.

### SIRT1 deacetylase activity assay

For deacetylase activity assay, samples were subjected to Fluor de Lys-SIRT1 assay. Briefly, samples were precleared using a 1:1 ratio of agarose-conjugated protein A bead slurry (Upstate Biotechnology), subjected to immunoprecipitation either by an antibody specific for sir2/SIRT1 (07-131; Upstate Biotechnology) or not using an antibody as a control, and then captured with agarose-conjugated protein A beads. Resuspended samples were added to a reaction mixture containing Ac-p53-fluorogenic-substrate (AK-555; Biomol Research Laboratories, Plymouth Meeting, PA, USA). Reactions were carried out at 37 °C for 25 min. Enzymatic activity was measured using a Spectramax Gemini fluorimeter (Molecular Devices, Sunnyvale, CA, USA). Comparable input SIRT1 protein was verified by immunoblotting.

## Supporting Information

**Fig. S1** Prolonged IGF-1 treatment induces cellular senescence in cancer cells. **Fig. S2** IGF-1 treatment does not affect the SIRT1–DBC1 interaction.
