# Supplementary material for: Insulin-like growth factor-1 regulates the SIRT1-p53 pathway in cellular senescence
Source: Aging Cell. 2014 Apr 30;13(4):669–78. doi: 10.1111/acel.12219 (PMC4118446; doi:10.1111/acel.12219)
Supplement: Supplementary file 1 — Fig. S1 Prolonged IGF-1 treatment induces cellular senescence in cancer cells. Fig. S2 IGF-1 treatment does not affect the SIRT1–DBC1 interaction. [file acel0013-0669-sd1.pdf]

Supplementary information

Fig. S1. Prolonged IGF-1 treatment induces cellular senescence in cancer cells. (A) IMR90 cells were serum-starved with 0.5% FBS for four days and then treated with 50 ng/mL IGF-1 every other day for six days. Cells were analyzed by flow cytometry at the indicated time points. (B) MCF7, HCT116($p53^{+/+}$) and HCT116($p53^{-/-}$) cells were serum-starved for four days and then treated with 50 ng/mL IGF-1 every other day for six days. Cells were then assayed for senescence-associated β-Galactosidase (SA-β-Gal) activity and photographed under a light microscope. (C) Senescent cells were quantified by counting the number of SA-β-Gal-positive cells over the total number of cells from four randomly selected fields of each cell culture plate. Results are presented as means and SE from two experiments. *: $P < 0.01$.

Fig. S2. IGF-1 treatment does not affect the SIRT1-DBC1 interaction. (A – B) MCF7 cells were serum-starved for 48 hours, and subsequently treated with 50 ng/mL IGF-1 for 12 hours. (A) Cells were collected and subjected to immunoprecipitation (IP) with a specific DBC1 antibody and then analyzed by immunoblotting (IB), as indicated. Two representative independent experiments are shown. (B) Cells were collected and subjected to IP with a specific SIRT1 antibody and then analyzed by IB, as indicated. One representative experiment

is shown. (C) MCF7, IMR90 and A549 cells were grown under normal growth conditions. Whole-cell lysates were subjected to western blotting, as shown.

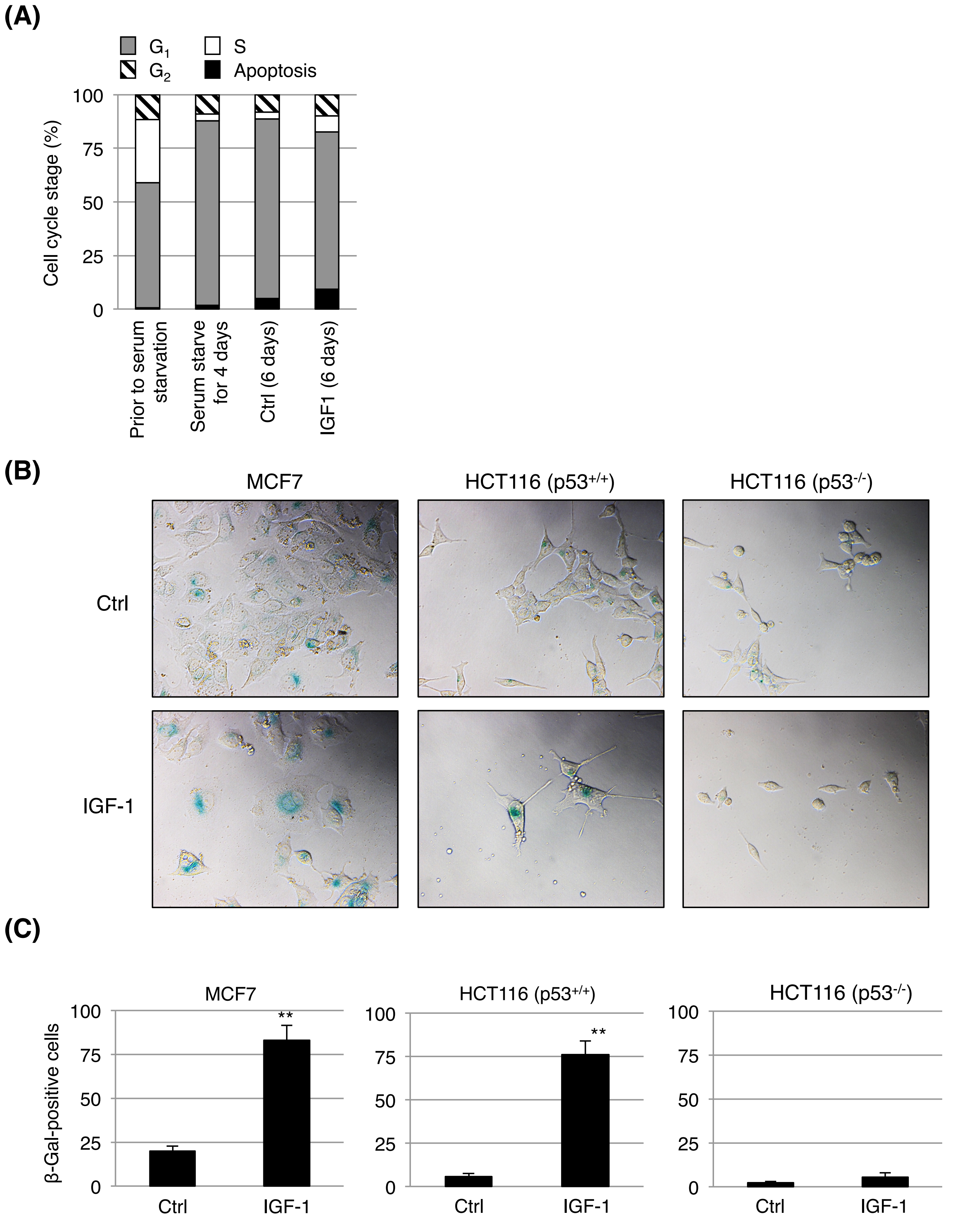

Supplementary Figure S1, Tran et al.

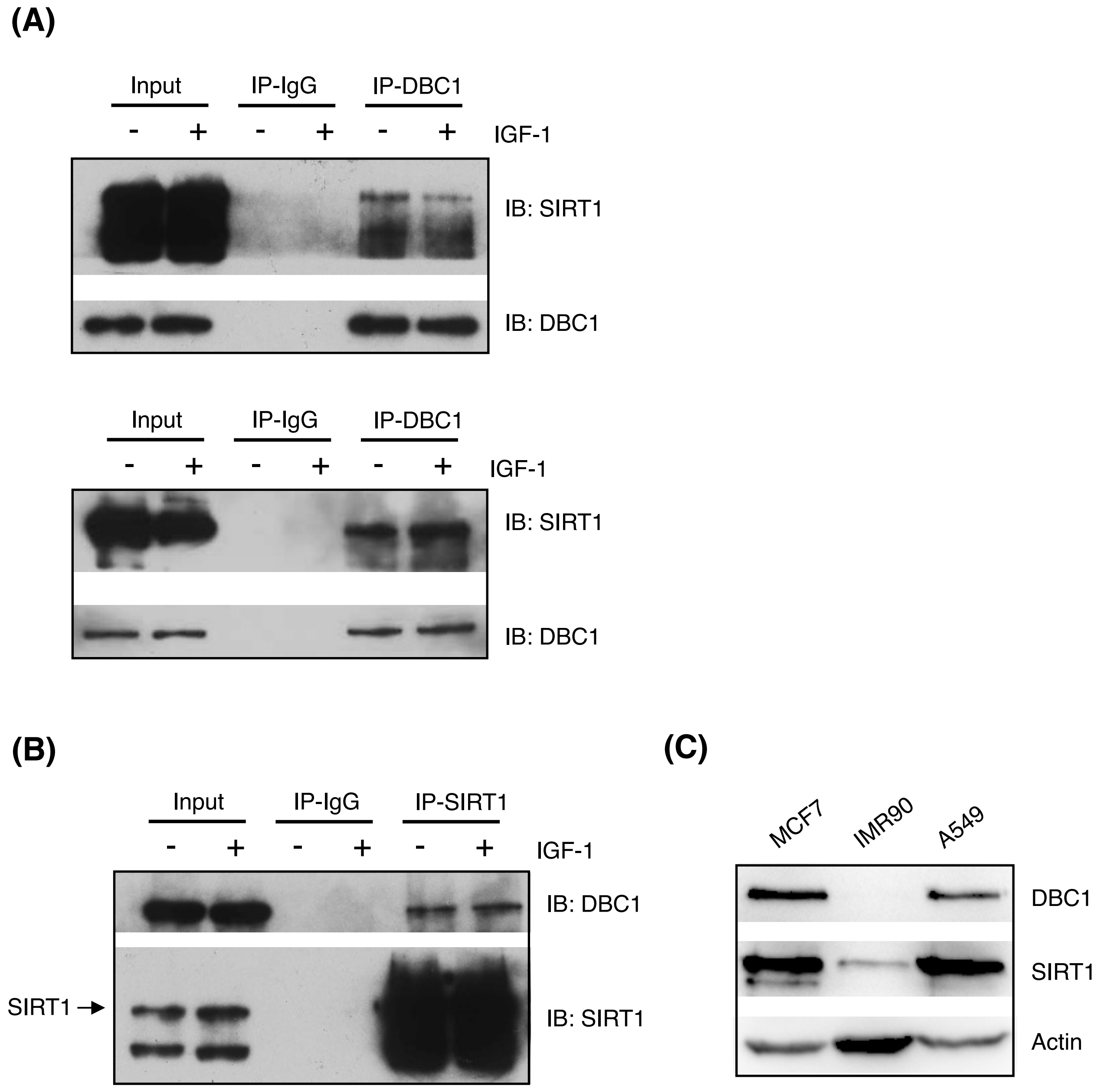

Supplementary Figure S2, Tran et al.
